# Mechanics of Undulatory Swimming in a Frictional Fluid

**DOI:** 10.1371/journal.pcbi.1002810

**Published:** 2012-12-27

**Authors:** Yang Ding, Sarah S. Sharpe, Andrew Masse, Daniel I. Goldman

**Affiliations:** 1 School of Physics, Georgia Institute of Technology, Atlanta, Georgia, United States of America; 2 Interdisciplinary Bioengineering Program, Georgia Institute of Technology, Atlanta, Georgia, United States of America; Princeton University, United States of America

## Abstract

The sandfish lizard (*Scincus scincus*) swims within granular media (sand) using axial body undulations to propel itself without the use of limbs. In previous work we predicted average swimming speed by developing a numerical simulation that incorporated experimentally measured biological kinematics into a multibody sandfish model. The model was coupled to an experimentally validated soft sphere discrete element method simulation of the granular medium. In this paper, we use the simulation to study the detailed mechanics of undulatory swimming in a “granular frictional fluid” and compare the predictions to our previously developed resistive force theory (RFT) which models sand-swimming using empirically determined granular drag laws. The simulation reveals that the forward speed of the center of mass (CoM) oscillates about its average speed in antiphase with head drag. The coupling between overall body motion and body deformation results in a non-trivial pattern in the magnitude of lateral displacement of the segments along the body. The actuator torque and segment power are maximal near the center of the body and decrease to zero toward the head and the tail. Approximately 30% of the net swimming power is dissipated in head drag. The power consumption is proportional to the frequency in the biologically relevant range, which confirms that frictional forces dominate during sand-swimming by the sandfish. Comparison of the segmental forces measured in simulation with the force on a laterally oscillating rod reveals that a granular hysteresis effect causes the overestimation of the body thrust forces in the RFT. Our models provide detailed testable predictions for biological locomotion in a granular environment.

## Author Summary

The sandfish lizard uses body undulation to propel itself within granular media (sand). Previously we developed a numerical simulation model consisting of an experimentally validated multi-particle model of the granular medium, and a sandfish model with prescribed body deformation (a traveling sinusoidal wave with parameters measured from biological experiment). We used the simulation to capture average swimming speed and compared predictions to our previously developed resistive force theory (RFT) for granular media. In this paper, we use the numerical model to perform more detailed analysis of the mechanics of sand-swimming in a so-called “granular frictional fluid”. These include center-of-mass kinematics, force distributions along the body, effects of body and head shape, power generation and dissipation. We discuss how these aspects of sand-swimming compare to those for swimmers (like nematodes and eels) in true fluids. We use the numerical model to reveal how transients during start-up in granular drag generates discrepancies between the simulation and the RFT predictions. The predictions from our models can give insight into locomotor capabilities, musculoskeletal structure and morphological features of sand-swimming animals. These results may also provide guidance for the design and control of sand-swimming robots.

## Introduction

Undulatory locomotion is widely used by organisms living in water [1], [2] and on the surface of the ground [3], [4]. However, thrust and drag forces can differ depending on the physics which govern the body-medium interaction. Small organisms, such as nematodes and spermatozoa, live in fluids where viscous forces dominate and inertia is negligible [1], [5]. Larger swimmers in water propel themselves with forces which arise from accelerating fluid. For terrestrial locomotion of undulatory crawlers like snakes and eels, frictional ground reaction forces provide thrust. Study of the mechanics of undulatory locomotion in varying environments advances our understanding of these organisms. The principles learned from the locomotion of organisms may also facilitate the development of robotic systems that can move efficiently in various environments [4], [6], [7].

Computational and theoretical tools have been used to obtain detailed understanding of the mechanics of swimming in fluids. The flow and pressure fields in fluids are well described by Navier-Stokes equations; however computing the force on the body of a swimmer can be a challenge in part due to unsteady flow conditions and limits of computing power. Theoretical models such as resistive force theory (RFT) [1] and Lighthill's elongated body theory [8] provide insights into the coupled dynamics between the hydrodynamics of media and the kinematics of the animal. Computer simulation which couples computational fluid dynamics (CFD), internal forces, and elastic structures enables examination of neuromechanical control hypotheses and analysis of morphological features beneficial to locomotion (e.g. [9]–[11]).

Granular environments such as sand-covered deserts, beaches, rain-forest soils and leaf litter are common habitats for terrestrial animals. A granular medium has a complex rheology since it can behave both like a solid or a fluid [12]: it remains static under stress until the yield stress is reached, after which it will flow and deform. Accurate equations at the level of the Navier-Stokes equations for fluids have not been developed for granular media. Further, flow visualization techniques in optically opaque granular materials are less advanced than those in fluids (e.g. Particle Image Velocimetry (PIV)). Nonetheless, some principles about the resistive force on an intruder moving within granular media have been revealed. At low speeds (quasi-static regime), effects of inertia are negligible and the resistive forces are dominated by gravitational (weight of media) and frictional forces. In this regime, forces are independent of speed and increase with depth [13]–[15]. Intruder shape has a small influence on drag force in granular media compared to that in high Re fluids [16].

**Figure 1:**
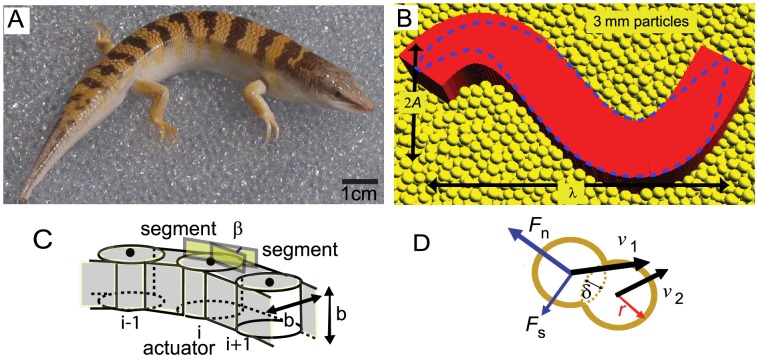
The sandfish lizard and the numerical simulation. A: A sandfish lizard (*Scincus scincus*) resting on 3 mm diameter glass particles. B: A simulated sandfish with a uniform body resting on simulated 3 mm particles. *A* and λ represent the amplitude and the wavelength of the single period sinusoidal traveling wave. Dashed purple line shows the outline of a tapered body. C: The elements of the sandfish model in the Working Model multibody simulation environment. The cuboid body segments are connected by actuators and *β* is the angle between two segments. D: Diagram of the empirical force relations used for particle interaction. The normal (*Fn*) and tangential (*Fs*) forces between two particles are calculated based on the relative speed *v* = *v*1−*v*2 and the virtual overlap *δ* between these two particles. Panels B, C, and D are reproduced from [21].

Recently we used high speed x-ray imaging to show that the sandfish lizard (*Scincus scincus*) (see Fig. 1A) uses body undulation to swim subsurface without the use of limbs [17]. Similar to some swimmers in fluids [2], [18], the body kinematics were well approximated by a single period sinusoidal wave traveling posteriorly (head to tail). The animal tended to use an amplitude (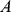) to wavelength (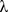) ratio 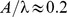. The ratio of the average forward swimming speed of the animal to the traveling wave speed, defined as the wave efficiency, was about 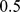. We developed a RFT model for granular media to predict the average swimming speed of the sandfish for varying undulation frequency. In the RFT model, the body was divided into infinitesimal segments, and the net force on the body and head in the forward direction (thrust and drag) was calculated by integrating forces on the segments. The force on each segment was assumed to be the same as the steady state force on a rod dragged with constant speed; these forces were determined empirically. We solved for the average swimming speed by balancing the thrust and drag.

The RFT model predicted wave efficiencies close to those observed in the animal experiments. It also predicted that the amplitude used by the animal resulted in maximum swimming speed, which was confirmed by a bioinspired sand-swimming robot [19]. However, the RFT model contained assumptions about the forces on the oscillating body segments and only motion in the forward direction was considered. In addition, the model was limited to the gait of a single-period sinusoidal wave. Therefore, a more accurate and flexible model was also developed by coupling an experimentally validated discrete element method (DEM) [20] simulation of the granular medium with a multibody simulation of the sandfish [21]. The simulation also predicted optimal average forward swimming speed at approximately 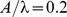. The functional forms of the speed vs 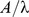 relationships were similar in simulation and RFT; however the RFT model systematically overpredicted speeds by 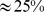.

Because our previous studies focused only on the average swimming speed, other aspects of the mechanics of swimming were not investigated [17], [21]. In this paper we use the previously developed DEM-multibody simulation model to examine more detailed swimming kinematics and the thrust/drag distribution along the body. The simulation reveals how features of granular resistive forces affect swimming performance, where and how the granular forces creates differences compared to swimming in fluids, and where the empirical force relations used in the RFT generate discrepancies between the RFT and the simulation. The simulation also generates biological predictions for energy generation and dissipation, which may have physiological and behavioral significance for sand-swimming animals.

## Materials and Methods

Our simulation consisted of a 3D discrete element method (DEM) simulation of the granular medium and a multibody simulation for the motion of the sandfish. We will refer to this multibody-DEM simulation as “the simulation”.

### Multibody simulation of the sandfish

The multibody simulation of the sandfish was implemented within the commercial software package Working Model 2D (Design Simulation Technologies). In the multibody simulation, the model sandfish was divided into 60 segments along its length (Fig. 1B). The segments were connected by actuators with one rotational degree of freedom so the body of the model sandfish could deform in its coronal plane (Fig. 1C). The actuators did not directly interact with the particles. The angle of each actuator was specified as a function of time (see [21] for simulation details) such that an approximate sinusoidal wave with constant amplitude traveled from head to tail:
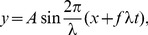
(1)where 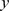 is the displacement from the midline of a straight animal, 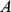 is the amplitude, 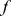 is the undulation frequency, 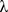 is the wavelength, 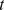 is the time, 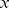 is the distance along a line parallel to the direction of the traveling wave, and the wave speed is 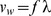. Because the model sandfish body was inextensible, the parallel position 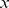 for a segment varied with time and undulation amplitude. Therefore, we used 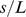 as an approximation of 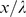, where 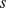 is the arc-length from the tail and 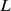 is the bodylength. We parameterized the position along the body as a number from 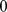 to 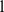, where 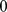 denoted the tail tip and 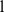 the snout tip. The model did not incorporate limbs since the sandfish placed its limbs along its sides during subsurface swimming [17].

We created two sandfish body plans (Fig. 1B): The first was a body whose width increased from 0.2 cm to 1.6 cm as the position along the body changed from 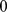 to 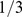 and decreased from 1.6 cm to 0.2 cm as the position changed from 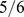 to 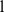 (see the dashed line in Fig. 1B). This allowed us to model a swimmer with the natural tapering of the sandfish body (in the coronal plane). We will refer to this as the “tapered” body simulation. To better compare with the resistive force theory (RFT) we also developed a sandfish model with uniform square cross section along the body and flat ends. We will refer to this as the “uniform” body simulation. The body length and height (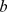) of the model sandfish were 12 cm and 1.6 cm, respectively. The mass of each segment was proportional to the cube of its width and the total weight of the simulated sandfish was 14 g for both body shapes. Based on previous animal observations [17], we set 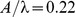. Since 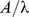 characterizes the shape of the sinusoidal wave and since it increases monotonically with increasing 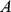, in the remainder of the paper we will refer to 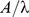 as the ‘amplitude’. We also performed simulations at smaller 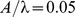 to examine force distributions along the body and the generality of the empirical force relations. The motion of the model sandfish was constrained within a plane. Unless otherwise stated, the plane was oriented horizontally to simplify our analysis. The model sandfish was placed initially at a depth such that the top surface of the model was 3.9 cm below the surface of the granular medium.

### Discrete element method simulation of the granular medium

The granular medium was simulated using our previously developed 3D soft-sphere DEM code. To compute particle-particle and body-particle interaction forces, we calculated the normal force [22], 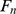, and the tangential Coulomb friction force, 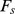, (see Fig. 1D) at each contact according to the force relations,
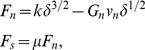
(2)where 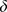 is the virtual overlap between contacting objects, 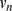 is the normal component of relative velocity, and 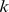 and 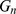 represent the hardness and viscoelastic constants, respectively. 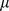 refers to the particle-particle (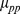) or body-particle (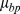) friction coefficient. Values of the coefficients are given in Table 1. We used a 50 ∶ 50 bi-disperse mixture of 3.4 and 3.0 mm particles to approximate the size distribution of the experimental granular medium consisting of 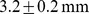 diameter (PD) glass particles [21]. As in [21], the simulation was validated by comparing the forces on a cylindrical stainless steel rod (diameter = 1.6 cm, length = 4 cm) dragged horizontally within the simulated medium and those from drag experiments within the real medium. In simulation, the container holding the particles was 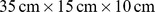 (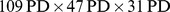) in volume and the initial volume fraction was set as 0.60 (see [23] for preparation details).

**Table 1:** Particle properties in simulation and in experiment.

	Experiment	Simulation
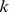 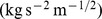	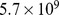	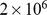
Restitution coefficient	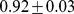	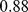
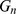 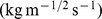	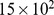	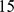
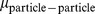	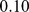	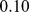
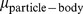	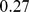	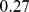
Density 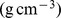	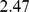	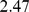
Diameter (mm)	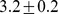	3.0 (50%) and 3.4 (50%)

PD is the average particle diameter of 3.2 mm.

To integrate the DEM simulation into the multibody simulation, the DEM code was compiled as a dynamic-link library and loaded by Working Model 2D. The DEM simulation calculated the forces between body segments and particles based on their positions and surface geometries. At each time step, forces on the segments were transferred from the DEM simulation to Working Model. Since we constrained the motion of the model sandfish to a plane, only the two force components within that plane were transferred to Working Model. Using the forces on the segments and prescribed actuation as inputs, Working Model calculated the motion of the model sandfish. At the end of each time step, the positions of the segments were transferred from Working Model to the DEM simulation. Before the simulation began, the model animal at its initial shape was placed in its designated plane and the particles that were in contact with or inside the animal were removed to avoid unrealistically large forces. Particles were allowed to settle for 0.3 seconds before the actuation began.

### Measurements in the simulation

Because the center of mass (CoM) trajectory of the model sandfish emerges from the interaction of the body wave with the resistive forces generated by the granular medium, the forward direction during swimming was not always parallel to the length of the container. For convenience, we therefore choose coordinates such that the x-axis was aligned with the forward swimming direction averaged over the entire motion and the y-axis was in the lateral (orthogonal to the forward swimming direction in the horizontal plane) direction. Since the waveform is a single period sinusoid with constant amplitude, the line that connects the tail and the head is parallel to the direction of the traveling wave. Therefore, we used the instantaneous angle of this line relative to the forward direction, 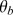, as a measure of the yaw motion. We observed significant fluctuations in forces in the simulations, similar to experimental drag forces in [24], [25]. To obtain a smoother force distribution on the body to compare to the force distributions from the empirical force relations, we averaged the forces from four simulations with granular beds prepared with the same method but different and random particle positions. We measured actuator torque and actuator power from the multibody simulation and segment force and segment power from the DEM simulation.

#### Empirical force relations

**Figure 2:**
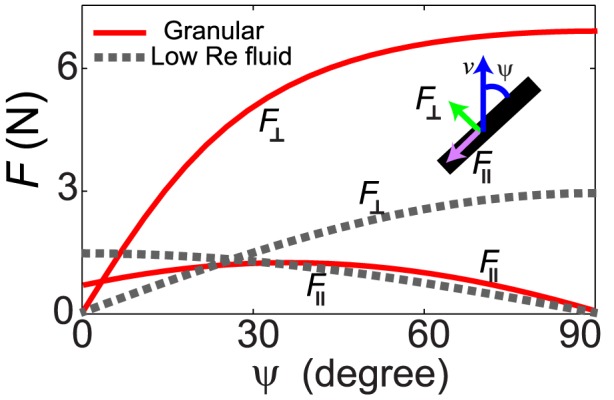
The empirical resistive force relations for the granular medium. The empirical force relations were obtained by dragging a rod with square cross-section (width = height = 16 mm, length = 40 mm) through 3 mm glass particles in simulation, at constant depth of 7.6 cm. The perpendicular (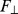) and parallel (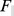) components of the surface forces are plotted as a function of the angle between the velocity direction and the rod axis (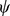), see inset. See text for the analytical expressions for 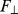 and 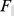. For comparison, 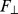 and 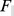 are calculated for a long thin ellipsoid in a low Re fluid by choosing a viscosity that gives comparable magnitude of 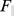; the low Re forces are plotted as dashed gray lines. Figure adapted from [21].

In the RFT (as in our previous work [17]) we assumed that the forces on the sandfish body were independent of speed and increased linearly with depth. Similar to the technique in [21], empirical force relations were obtained by measuring steady-state forces (decomposed into a perpendicular component 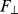 and a parallel component 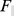) on a square rod. These were well described by the following fitting functions (see Fig. 2) [21]:
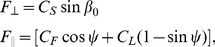
(3)where 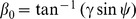; 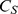, 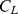 and 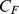 are fitting parameters with values listed in Table 2 in the Appendix. To evaluate the accuracy of the empirical force relations, we calculated forces on the model sandfish predicted by the empirical force relations and compared them with forces directly measured from simulation. The angle 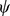 for a segment in simulation was found by calculating the angle between its velocity and the axis direction 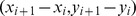, where 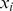 and 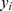 are the 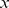 and 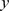 components of the 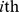 segment. The forces on the body were approximated using the parameters from the forces on the long surface of the rod, and the forces on the head were approximated with the parameters from the forces on the end cap of the rod. The forces were scaled assuming that the forces were proportional to cross-sectional area and depth. The cross-sectional area of the head (in the transverse plane) was 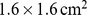 and the cross-sectional area of each segment (in the sagittal plane) was 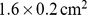.

**Table 2:** Fitting parameters for the analytical functions approximating 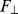 and 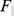.

Fitting parameter	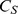 (N)	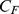 (N)	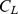 (N)	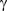
3 mm particles (body)	5.57	2.30	−1.74	1.93
3 mm particles (head)	19.52	1.24	−0.99	0.14

### Animal experiment

To compare the segment motion predicted by the model with those from animal experiments, we placed opaque markers along the midline of an animal, and the trajectories of the markers were obtained from high speed x-ray video of the sandfish lizard swimming in 3 mm glass particles (n = 4 runs, N = 2 animals, see [26] for experimental details). All experimental procedures were conducted in accordance with the Georgia Institute of Technology IACUC protocol number (A08012) and Radiation 159 Safety protocol (X-272). Because the height/diameter of the animal body decreases rapidly beyond about 1.2 snout-to-vent length (SVL) and we estimate this region generates little force. To compare experiment and simulation we approximated the effective total body length of the animal (in terms of force generation) as 1.2 SVL to compare experiment and simulation.

## Results

### Kinematics

**Figure 3:**
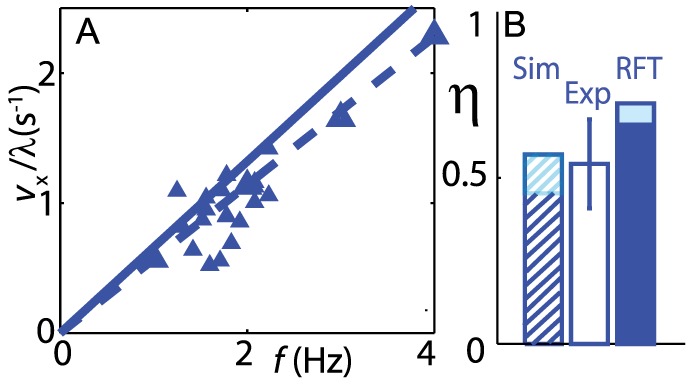
Comparison of swimming performance in experiment and models. A: Average forward swimming speed versus undulation frequency in 3 mm particles. Solid symbols correspond to biological measurements, and the solid and dashed lines correspond to the RFT (for a uniform body) and simulation (for a tapered body) predictions, respectively. B: Wave efficiency (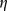), defined as the ratio of the forward swimming speed to the wave speed, measured from biological experiment (the slope of 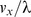 versus 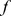 in (A)), simulation and RFT. For the RFT (solid bar), the lower and upper limits of the 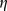 deviation correspond to maximum (flat head) and 30% of the maximum head drag, while the simulation (hatched) corresponds to the uniform body and tapered body shapes, respectively. In simulation, 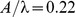 and 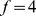 Hz. Figure adapted from [21].

In [21] we reported that the average swimming speed as a function of frequency from simulation was in accord with experimental results. The wave efficiency (average swimming speed normalized to traveling wave speed) 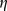 predicted by the simulation agreed well with experiment (Fig. 3). The RFT over-predicted the wave efficiency by about 20%. In the following sections we compare more detailed body and segment kinematics in biological measurements and simulation.

#### Body kinematics

**Figure 4:**
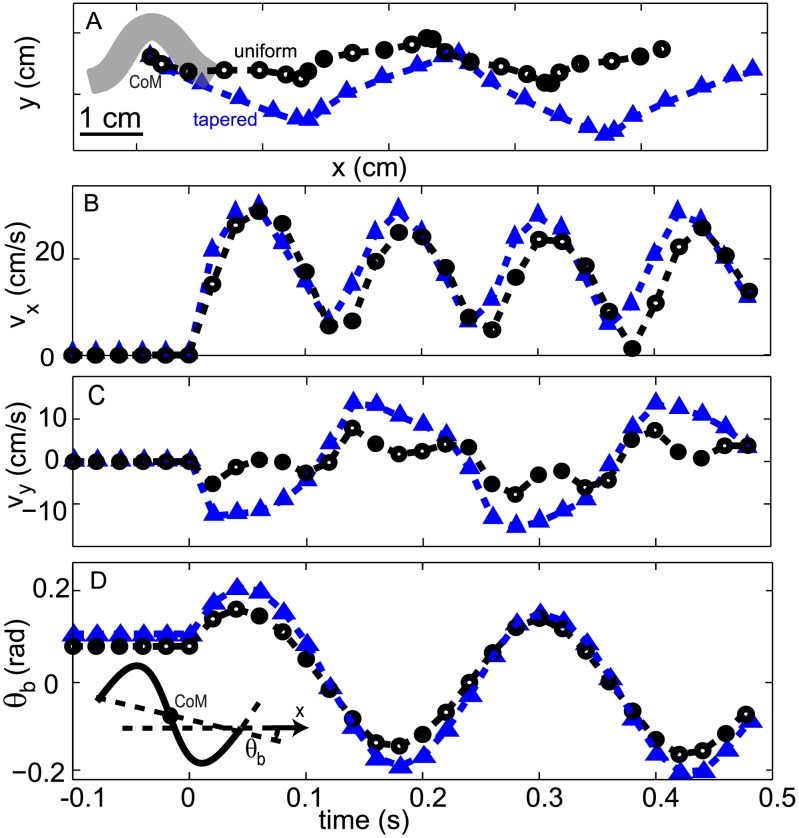
Body kinematics of the sandfish in the simulation. A: CoM trajectory. Gray region shows the configuration of the uniform body at 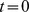 s (not to scale). The (B) forward velocity, (C) lateral velocity and (D) body orientation as a function of time. Inset: Diagram showing calculation of the center of mass (CoM) and yaw angle (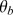). Black circles represent uniform body and blue triangles represent tapered body. Actuation began at t = 0 sec. 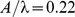, 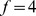 Hz.

For both the uniform body and tapered body, we measured three degrees of freedom of the overall body motion: forward motion, lateral motion and yaw motion (rotation). The dominant motion was in the forward direction, and the forward speed oscillated with a peak-to-peak magnitude about 60% of the average speed. The frequency of the speed oscillation was twice of the undulation frequency. The lateral velocity and displacement of the CoM were small compared to those in the forward direction, as shown in Fig. 4A & C. Oscillation about the yaw axis with maximal angular excursion of about 9 degrees was observed as the model sandfish swam forward. For the tapered model, the oscillation amplitude of the forward speed was slightly smaller and the oscillation amplitude of the lateral speed was larger. In both body shapes the forward speed as well as other velocity components,reached the steady state pattern within 1/4 cycle (Fig. 4). The absence of a transition period, in contrast to transitions of a few cycles to achieve steady state swimming in high Re fluid (e.g. [11]), indicates that the inertia of the body and surrounding material were negligible. Direct comparison to the CoM motion in experiment was not made due to the uncertainty of mass distributions of the animal and its tail position in the x-ray videos.

#### Segment kinematics

**Figure 5:**
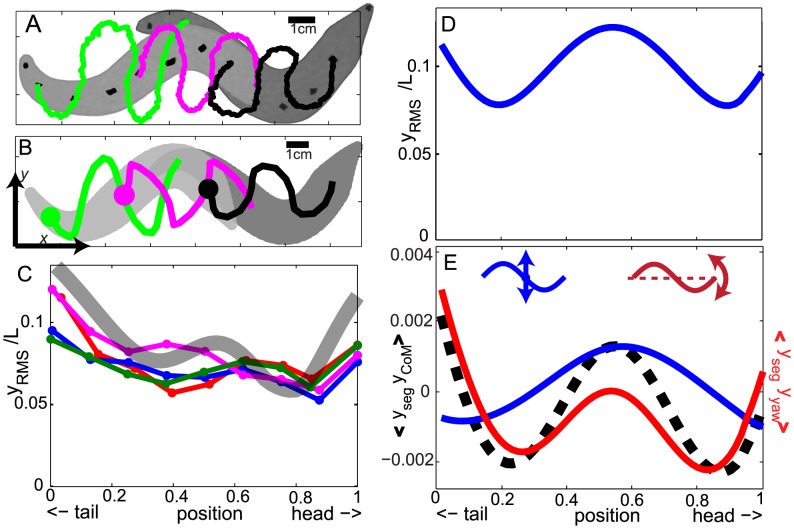
Segment kinematics of the model sandfish. Trajectories of segments near the head, middle of the body, and the tail from both experiment (A) and simulation (B) are represented by black, magenta, and green lines, respectively. The markers in experiment are located at 0.13, 0.50, and 0.87 of the effective body length and the segments at 0.16, 0.50 and 0.84 of the total body length (defined as the length from snout to tail tip) are chosen as counterparts in the simulation with a tapered body. The light gray regions indicate the body position at an earlier time and the dark gray regions indicate the body positions at 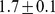 cycles later. C: The RMS of the lateral displacement of a segment normalized to the effective body length as a function of position on the body from experiments (colored lines and symbols) and a simulation with a tapered body, an entry angle of 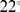, and an amplitude of 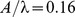 (thick gray line). The data is from two animals with contributions of 1 (green) and 3 (other color) runs. D: The RMS of the lateral displacement of a segment normalized to the total body length as a function of position on the body of the model sandfish. E: The correlation between the lateral motion of segments and the lateral motion induced by the CoM motion (the blue curve and the left inset) and yaw motion (the red curve and the right inset) of the body. The data is from the same simulation for panel A. The dashed black line is the sum of the blue and red lines. In (D) and (E), the model sandfish swims in the horizontal plane with a uniform body at 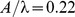 and 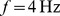.

Since the motion of a segment on the swimmer is the combination of overall body motion and prescribed segment motion in the body frame (body deformation), the segment trajectories in the lab frame may differ from the prescribed segment motion–a traveling wave with constant amplitude–and depend on the position along the body. Segment trajectories from simulation showed a pattern similar to those from experiment (Fig. 5A & B). To better compare simulation results with the experimental data shown in Figure 4, we used the tapered body inclined at 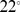 with respect to the horizontal and 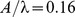. The incline angle and 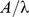 were within the range of reported values (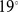 to 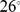 and 0.1 to 0.3, respectively) from experiment [17], [26].

To characterize the undulatory motion of the segments, we calculated the root mean square (RMS) of the lateral displacement 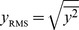 for each segment. As shown in Fig. 5C &D, 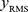 displayed a “w”-shaped pattern as a function of position along the body in both simulation and biological experiment. The amplitude was larger near the middle and ends and displayed two local minima near the 0.2 and 0.8 points of the total body-length. The smaller average magnitude of 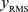 and a trend of increasing magnitude from the head to the tail from biological experiment were captured by the tapered simulation with a smaller amplitude 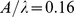 and an entry angle of 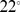.

The pattern of the lateral displacement is a result of the coupling between the prescribed lateral motion of the segments and the motion of the body. Since the forward motion of the body is orthogonal to the lateral motion of the segments (in the lab frame), only the lateral and yaw motions of the body must be considered. Therefore, to identify how the pattern was generated, we examined the correlation between the lateral displacement of a segment (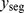) to the lateral displacement of the CoM (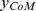) and yaw motion (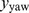) (see Fig. 4B). 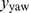 was approximated as 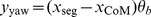, where 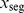 is the position of the segment in the forward direction. We quantified the correlation using the product of the lateral displacements averaged over a cycle: 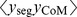 and 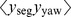. The positive/negative sign of 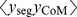 and 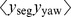 indicated the CoM and the yaw motion enhanced/reduced the lateral motion of a segment. The shape of the two correlation plots indicated that the CoM motion was responsible for the enhancement of the lateral motion of the central segments, while the two minima near 0.2 and 0.8 of the body length were caused by the yaw motion. We hypothesize that the increasing amplitude towards the tail observed in biological experiment is a result of the decreasing resistive force towards the tail, because: (1) resistive forces increase with depth within granular media, (2) the depth decreases from head to tail when the entry angle is nonzero, and (3) the increasing amplitude of lateral displacement only appears in the simulations with entry angles of 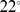.

### Forces

#### Force from simulation

**Figure 6:**
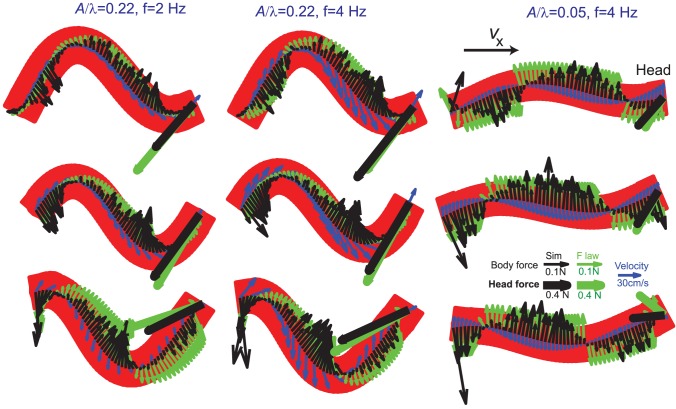
Snapshots of reaction forces on the model sandfish during swimming. Black, green, and blue arrows represent forces measured in uniform body simulation, forces predicted by the empirical force relations, and velocities, respectively. For visibility, only every 3rd segment velocities are shown for 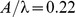 and the head drag is scaled by a factor of 0.25 and drawn in thick lines. Snapshots in the middle column were taken at 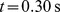, 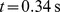 and 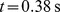; snapshots with the same phases were chosen for the other two columns. The values below the arrows in the legend indicate the magnitudes of force and velocity corresponding to the length of their respective arrows. All diagrams show forces for a uniform body.

**Figure 7:**
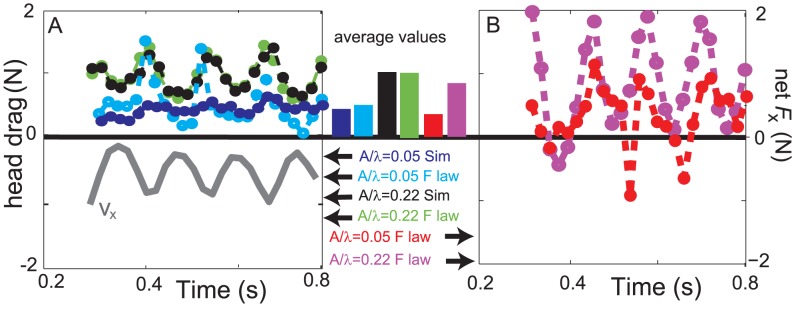
Comparison of head drag and net force on the model sandfish from the simulation and empirical force relations. A: Head drag (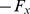 on the head) as a function of time for 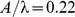 (green represents the empirical force relations and black represents simulation) and 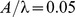 (cyan represents the empirical force relations and blue represents simulation). The forward speed for 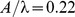 is re-plotted from Fig. 3B as the gray line to show relative phasing. B: The net force on the body (including head) in the forward direction as a function of time for 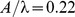 (magenta) and 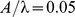 (red). Bars between (A) & (B): The average values of the net forces and the head drag are given in corresponding colors. Uniform bodies were used in the simulations. 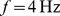.

In simulation, the net resistive force on every segment was calculated from the grain forces acting on the segments. The force on each segment pointed opposite to (but not co-linear with) the velocity of that segment (see Fig. 6). For 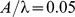, the forces on the body were mainly lateral and for the typical animal 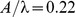, the larger angle between the segments and forward direction resulted in a larger net thrust force on the body. For both amplitudes, we observed substantial head drag (the thick lines in Fig. 6), which (along with body drag) was overcome by the thrust generated by the body. The head drag predicted by the empirical force relations showed a similar pattern as that from the simulation, and the average values of the head drag from empirical force relations quantitatively agreed with the simulation (see Fig. 7A). The variation in head drag was in antiphase to the variation of the forward speed (dashed grey line in Fig. 7A), which implies that the variation in forward speed was dominated by the variation of the head drag. In the simulation with 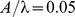, both the head drag and the thrust from the body were approximately 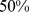 smaller than the forces in the simulation with 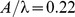.

#### Force comparison

Overall, the empirical force relations correctly predicted the direction and the spatial pattern of thrust and drag forces on the model sandfish as compared to simulation (Fig. 6). The forces on the model sandfish did not change significantly for frequencies less than 4 Hz (Fig. 6A & B), which is consistent with the RFT assumption that force is independent of speed, and is in accord with rod drag data in experiments [17]. However, we observed significant discrepancy in the magnitude of the forces on the body between those from empirical force relations and those from the simulation. The magnitudes of the forces measured in simulation were in general smaller. The differences were largest near the maximum lateral excursion, where velocity (and force) reversal occurred in the lateral direction.

As shown in Fig. 7B, because of this overestimation of thrust, the net forward force calculated from the empirical force relations was larger than zero, the value assumed in the RFT model and the average value observed in simulation at steady state. Since the empirical force relations were used in the RFT model, this overestimation of thrust force would also occur in the RFT model. Therefore the overestimation of the force magnitude resulted in the overestimation of speed in the RFT model. Such an overestimation of speed was observed in [21], where RFT speeds were 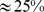 higher than the simulation.

#### A transient effect in granular drag force

**Figure 8:**
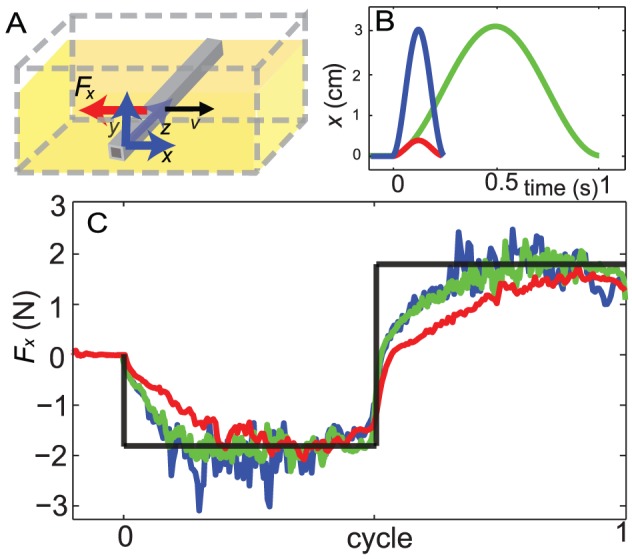
Drag forces on an oscillating rod. A: Schematic diagram of the simulation. The rod oscillates horizontally and normal to its axis. Rod width = 1.58 cm. B: The lateral displacement of the rod as a function of time. C: The resistive force in the lateral direction as a function of time. Blue, green and red lines represent the data from simulation with parameter sets (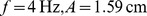), (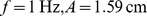), and (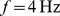 and 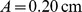), respectively. The black line represents the prediction from the empirical force law.

The smaller force magnitude near the reversal of lateral motion in simulation compared to that from the empirical force relations suggested the origin of the discrepancy might be a transient effect during granular drag. To investigate the transient force during velocity reversal, in simulation we measured the resistive force on an oscillating rod (see Fig. 8A). The rod was 10 cm long, 1.6 cm wide and had a square cross section. The displacement of the rod normal to its axis was prescribed as a sine function in time to mimic the undulatory motion of a segment on the animal body. We chose frequencies 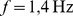 and amplitudes 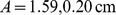, which corresponded to the undulation amplitudes of 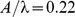 and 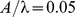 (see Fig. 8B).

We found discrepancies between the simulation and the empirical force relations in accord with our transient hypothesis: Based on the empirical force relations, the oscillatory motion should generate a drag force as a function of time with a square wave shape (the black line in Fig. 8C), since we assume the force only depends on the motion direction. In the simulation however, when the rod started moving in a new direction, either from rest or the opposite direction, a significant portion of the cycle (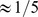 for the amplitude corresponding to 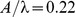) was required for the force to increase to its steady state value. Consistent with the assumption that forces are independent of speed, this discrepancy did not change significantly with different frequency (the blue line and the green line). However, the amplitude of the oscillation significantly affected the force. For the smaller amplitude case, the rate of the increase in magnitude was smaller and a larger portion of the cycle was in the transient region (the red line). A similar transient weakening effect has been observed and studied in a cyclically sheared granular medium [27]; the underlying physics of this effect may be related to changes in geometric structure among particles due to preparation methods or previous disturbances [27], [28].

The rod drag simulation implies that the transient effect associated with lateral displacement plays an important role in the overestimation of the forces. Note that the lateral motion of a segment in relation to its local segment axis is not the same as the lateral motion in relation to the forward direction of the body. As shown in Fig. 6, for the smaller amplitude case, the lateral velocity of a segment relative to its local segment axis is nearly the same as the lateral velocity in relation to the forward direction of the body, since the segment axes are nearly aligned with the forward direction. For the larger amplitude case, the velocity of a segment in the direction normal to the segment axis is on average only 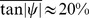 of the total velocity. In the extreme case such that the swimming speed is equal to the wave speed, every segment moves in the same direction of its axis and hence there is no motion normal to segment axes. Therefore, this transient weakening effect on the lateral forces may still influence (reduce) thrust generation at large amplitudes. This explains in our previous study [21] the over-prediction of force by the empirical force relations and the over-prediction of speed by the RFT model at all amplitudes. In addition, these results imply that in granular media, the transient effect is more important than the segmental interaction effects, which contribute significantly to the discrepancy between true fluid RFT and experiment [29].

#### Body shape effect

**Figure 9:**
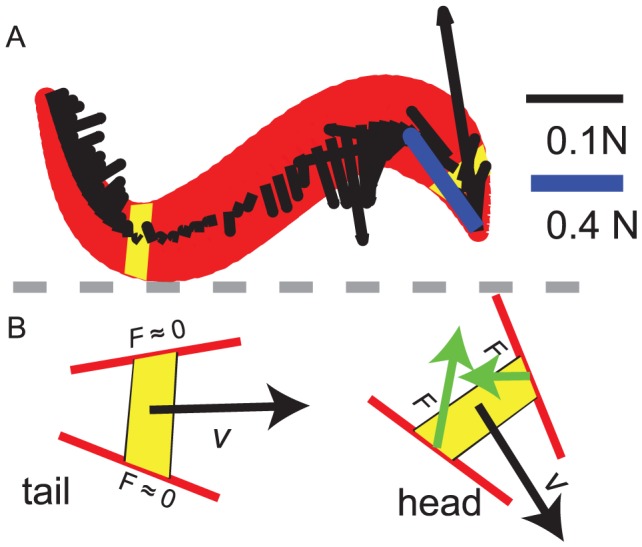
The effect of body taper on the force on a segment. A: A snapshot of the resistive forces on segments (black arrows). The blue arrow represents the head drag (net force on the tapered portion) with a different scale. B: Diagram of the forces on the segments in the tapered body regions near the tail (left) and near the head (right) when the velocities are nearly aligned with the mid-line of the segments. Corresponding segments are highlighted with yellow color on the body in panel (A). 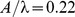, 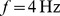.

The thrust and drag distributions on the tapered and the uniform bodies were similar, see Fig. 9A. Nevertheless, the pattern of force along the tapered body showed some differences compared to the uniform body. Because the orientation of the surface on the tapered part of the body was not parallel to the axis of the body, forces on both sides of the head contributed to the net force on the head (see Fig. 9B). The head drag for a tapered head, calculated by summing the drag on the tapered segments on the head and shown with thick blue line in Fig. 9A, was on average 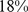 smaller compared to the drag on the blunt head. For the tapered tail, the velocity of a segment occasionally aligned with its axis. Therefore, the segments experienced nearly zero drag or thrust forces (Fig. 9B). The average swimming speed of the model sandfish with the tapered body was 20% higher than the uniform body at 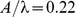.

### Actuator Torque

**Figure 10:**
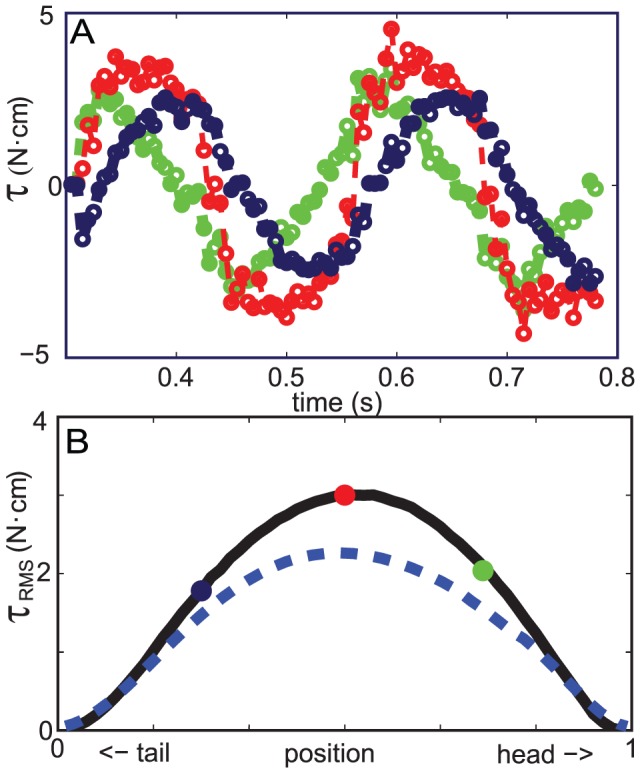
The torque generated by actuators of the sandfish model in simulation. A: The torque generated by actuators at 0.25, 0.5, and 0.75 of the body (represented by green, red, and dark blue symbols and lines, respectively) as a function of time. B: RMS magnitude of the torque of an actuator as a function of position on the body. Large filled circles indicate the RMS of the torque curves in panel (A) with the same color scheme. The solid black line in panel B and data in panel A are from simulation with a uniform body and the blue dashed line in panel B is from a simulation with a tapered body. 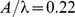, 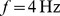.

The actuator torque 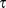 was measured directly in the simulation. It oscillated at the same frequency as the body undulation, but both the magnitude and phase varied along the body (Fig. 10A). To quantify the magnitude of 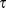, we measured the root mean square value 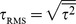. For both the tapered body and uniform body, 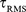 was maximal near the center and decreased symmetrically towards the ends of the body, resembling a bell shape (Fig. 10B). Because inertia during sand-swimming is negligible (see the body kinematics subsection), the actuator torque should equal to the sum of torques from all segments on either side of the body. Since the segment motion and force are similar along the body (excluding the head), the moment arm and integration length largely determine the magnitude of torque. From this argument, it is not surprising that the largest torque is observed near the center, where the distance to its nearest end is maximal.

### Power

**Figure 11:**
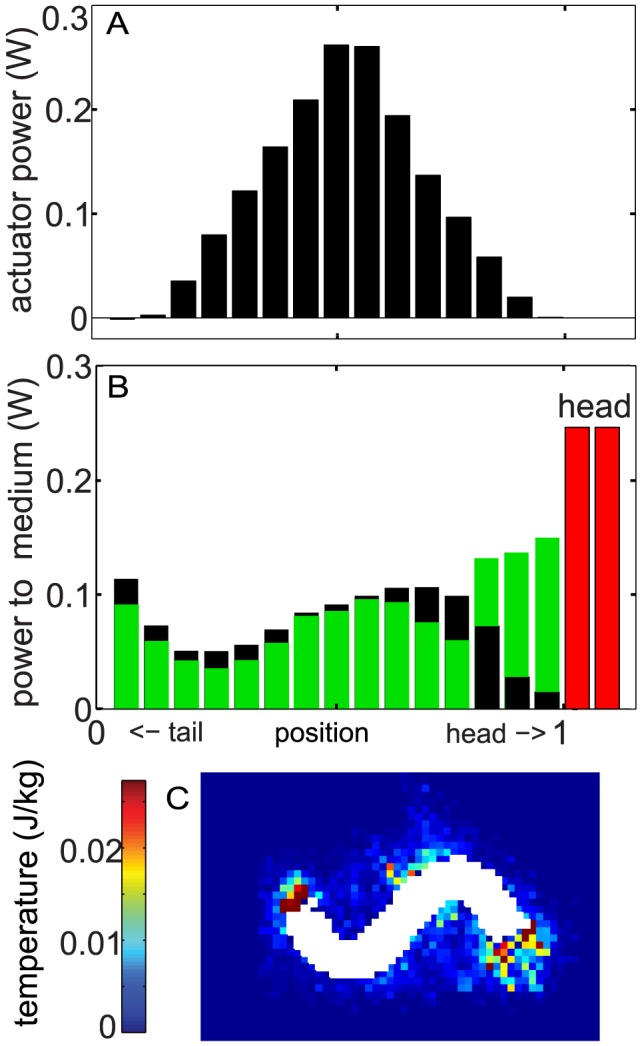
Spatial distribution of actuator power and power delivered to the granular medium. A & B: Each bar represents a 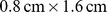 cross-sectional area (half cross-sectional area of the head) along the body or on the head (head area = 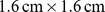). The black bars represent areas on the uniform body and red bars represent areas from the blunt head. The green bars represent areas on the tapered body. C: The granular temperature (see text for the detail) calculated from particles within cells with dimensions of 0.3 cm (W) by 0.3 cm (L) by 1.6 cm (H) for a uniform body. 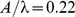, 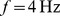.

**Figure 12:**
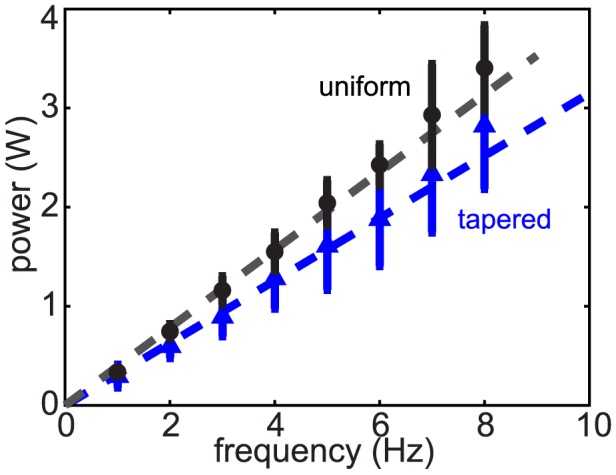
The total power generated by the actuators as a function of undulation frequency. Black circles represent uniform body and blue triangles represent tapered body. Dashed lines indicate a linear relationship between power and frequency. Fits are constrained to go through the origin and have slopes of 0.39 J (uniform) and 0.32 J (tapered). Averaging was done over an integer number of cycles and approximately 1 s of time. Error bars indicate the standard deviations of the fluctuations. 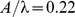.

Energy was generated from the actuators and dissipated to the medium through the resistive forces on the segments. The power output from the actuators was directly measured from the simulation and the power dissipated to the medium from body segments was calculated as the product of resistive force and segment velocity. To compare the power on the body with the power on the head, we use a histogram representation (Fig. 11A & B) and chose an integration area for power that is half of the area of the flat head (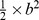). The total power can be calculated by either summing the actuator power (generation) or the segment-granular interaction power (dissipation); the differences of the power calculated from the two methods were within the fluctuation of the power as a function of time. The actuator power along the body also displayed a bell-shape distribution (see Fig. 11A): the central actuators generated most of the power and the actuators near the ends generated nearly zero or even slightly negative power (see the leftmost bar). We found that about 30% of the power was used to overcome the head drag for 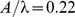. On the body, the distribution of power delivered by segments to the medium showed a “w”-shaped pattern (Fig. 11B) similar to the amplitude of lateral undulation of the segments. The power was enhanced in the middle and two ends and was reduced near the 0.2 and the 0.8 locations along the body. Although the total power consumption of the model sandfish with a tapered body was approximately 20% smaller than that with a uniform body, the power distribution on the tapered body showed a similar pattern. Similar to the uniform body case, the head drag consumed about 30% of the total power for the tapered body. The total power increased linearly with frequency within the biologically relevant range (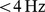); slight deviation was observed at frequencies higher than about 6 Hz (Fig. 12).

Inside the granular medium, the energy was dissipated due the dissipative interaction forces resulting from collisions and relative motion between particles. The granular temperature, which measures the deviatoric portion of the velocity field, can be used as an indicator of energy dissipation and fluidity of the local material [12]. As in [30] we calculate 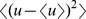 to measure the local granular temperature field, where 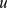 is the particle velocity and “
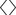
” denotes averaging over cells (see the caption of Fig. 11 for details) at the same depth of the model sandfish. As shown in Fig. 11C, high temperature regions appeared only in the vicinity of body and decayed to nearly zero within a distance comparable to the diameter of the body. Consistent with the distribution of the power dissipation, the granular temperature was highest near the head and the tail. The localized high temperature regions indicate the swimmer only fluidizes a limited volume of granular material and energy is dissipated locally.

## Discussion

The simulation provides detailed predictions for force distributions, torque requirements, and energetic costs associated with sand-swimming by the sandfish lizard. We next discuss the implications of these predictions on aspects of the morphology, control and physiology of the sandfish and possibly other sand-swimming animals. We also compare the predictions from the sand-swimming simulation to the results from undulatory locomotion in other environments to reveal mechanical features that are either common to undulatory swimming or particular to granular media.

### Force

#### Effect of body slenderness

In our simulations the head drag consumed a significant portion of energy generated by the body. However, drag forces in granular media are not sensitive to the shape of an intruder [16]. Therefore the head drag acting on the sandfish was mainly determined by the projected head area. However, since the swimming performance is determined by the balance of thrust and drag, the head drag plays a significant role in setting swimming speed. This head drag is overcome by thrust from the body and therefore, the locomotion ability depends on the ratio between the body and head areas, 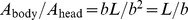. This implies that sand-swimming animals with a longer body may overcome the head drag more easily and reach a higher speed at the same frequency. For example, the shovel nose snake (*Chionactis occipitalis*) has a body-head area ratio of 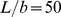, approximately 5 times that of sandfish. Preliminary studies show that the snake moves subsurface rapidly and with a higher wave efficiency than the sandfish lizard. Quantitative analysis of the subsurface kinematics of a diversity of slender fossorial snakes [31], [32] could test our model predictions.

#### Inertial force

Since the granular force is insensitive of speed at low speed due to its frictional origin at the grain level, total power required to swim increases linearly with frequency for low frequencies. At higher frequencies (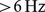) (see Fig. 12), the slight deviation from a linear relation between power and frequency implies that inertial forces due to acceleration of granular material become non-negligible. To test this hypothesis we estimated the magnitude of the inertial force due to acceleration of material around the body and compared it to the measured total force, which included friction. If we take the force on an area of 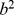 (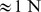) on the body as an example, the contribution from inertial force can be estimated as 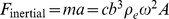, where 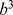 is the characteristic volume of granular material accelerated by the body, 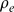 is the density of the medium taking into account of the voids between the particles, 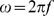 is the angular velocity, and 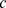 is a geometric coefficient. Assuming the shape of the accelerated volume is a cube, then 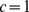. The formula yields 0.07 N for 4 Hz and 0.28 N for 8 Hz, which is about 7% and 28% of the total force. Because 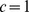 is unlikely to be exact and we used the maximum total force, this is a rough approximation. Nevertheless, this estimate and the power-frequency curve (see Fig. 12) from simulation both indicate that material inertia becomes non-negligible at frequencies 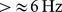. Note that the scaling of the non-inertial forces and the inertial forces are different: non-inertial forces increase with depth while the inertial forces scale as 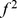 and do not depend on depth. This implies that the inertial force may dominate when the animal is closer to the surface and undulates at a high frequency. However, at typical animal undulation frequencies (less than 3 Hz), the depth at which the inertial forces could have significant contributions (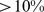) is less than the diameter of the animal. Therefore, based on our calculations, inertial forces become negligible as soon as the animal is subsurface.

### Power generation

To investigate energetic demands of swimming in the granular medium for a sandfish, we estimate the maximum power output from muscle and compare it to the predicted mechanical power from simulation. Assuming the mass of the animal is 14 g, 50% of the body mass is muscle, and the maximum muscle power is 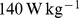 [33], the maximum mechanical power output can be estimated as 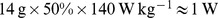. In simulation, this power corresponds to a frequency of about 2 Hz at the depth of 4 cm for a tapered body. Considering the animal has been observed to swim in the laboratory at a maximal frequency of 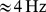 and a depth of nearly 10 cm, power required for sand-swimming may be near the limit of sandfish muscle. Therefore, the muscle power might be the limiting factor of the swimming speed and depth.

Predictions of the required mechanical power for swimming might be tested with animal experiments. Muscle power can be determined in sandfish *in vitro* using the work-loop technique [34]. With this method, muscle is attached to a force transducer and subjected to sinusoid length changes while applying a stimulus. These stimuli parameters can be determined from *in vivo* measurements [26]. Also, a large stimulus can be used to determine the peak power produced in muscle segments along the length of the body. Furthermore, metabolic consumption can be compared in sandfish by measuring oxygen consumption as well as lactate and glycogen concentration in muscles [35], [36].

### Cost of Transport

The mass specific mechanical cost of transport (CoT) is defined as 
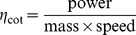
. By further dividing this by gravity, a non-dimensional cost of transport can also be calculated: 
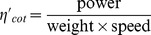
 [37], which facilitates comparison among different swimmers, burrowers and diggers. Since for the simulation of the sandfish both the power and the speed increased linearly with the frequency for 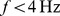, frequency did not affect the mechanical CoT in this range. We compared the predicted mechanical CoT from the model with other forms of locomotion, including the CoT of running and sand-burrowing of the Namib moles, sand-burrowing of the Atlantic razor clam, mud-burrowing of the polychaete, as well as others (see values and references in Table 3). The predicted 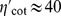 from our sandfish model (with a tapered body) is comparable to the CoT of the sand-burrowing of the razor clam and between the metabolic CoT for running and sand-burrowing of Namib moles. The predicted CoT of sand-swimming is much larger than the CoT of swimming in high Re fluids and smaller than the CoT of swimming in low Re fluids. Note that CoT generally increases with decreasing body weight and low Re swimmers in water are much smaller than the sandfish lizard [37].

**Table 3:** Comparison of cost of transport.

Animal	Sandfish	Mole	Mole	Razer Clam	Lizard	Eel	Nematode	Polychaete
Locomotion mode	sand-swimming	running	sand-burrowing	sand-burrowing	running	swimming	swimming	mud-burrowing
Metabolic CoT	N/A	15 [44]	400 [44]	N/A	3 [45]	0.4 [46]	N/A	N/A
Mechanical CoT	40	N/A	N/A	40 [47]	0.1 [45]	0.2 [48]	100 [49]	3 [50]
Mass (kg)	0.01	0.02 [44]	0.02 [44]	0.05	0.01 [45]	0.01 [48], 0.7 [46]	1e−7 [51]	0.005 [50]

Values above are typical values and are given to one significant digit. “N/A” indicates the value was not found in the literature.

### Phase between the angular velocity and the torque of an actuator

Because the actuator power is the product of angular velocity (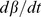) and torque (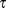), the sign of the power is an indicator of the phase difference between the angular velocity and torque of the actuators. The nearly zero or slightly negative actuator power near the tail and head indicates that the angular velocity is in antiphase with the actuator torque while the positive power indicates the two variables are in phase. The sign of power also implies that the phase between actuator torque and angular velocity varies along the body. Variations of the relative phase between curvature and muscle activation (measured through EMG), often referred to as “neuromechanical phase lags,” have been observed for both aquatic and terrestrial undulatory animals [9], [11], [38], as well as in the sandfish [26]. Since muscle activation is closely related to torque generation, and the curvature is related to angular velocity, our results suggests that the torque required by undulatory swimming in sand may (partially) account for the phase lag observed on the sandfish. Body stiffness and elasticity, which are determined by muscle and passive elements such as tendons, play important roles during swimming in fluids and have been shown to affect the phase shift between the curvature and muscle activation [11], [39], [40]. Future studies will quantify the body properties of the sandfish and include these in a more comprehensive model to study the neuromechanical control of the sandfish during swimming in sand.

### Comparison of undulatory locomotion in various environments

It is instructive to briefly compare the mechanics of undulatory swimming in a granular frictional fluid to those in Newtonian fluids like water and undulation on hard frictional ground. Similar to swimming in true fluids, in granular swimming the coefficient for the normal component of the resistive force is larger than the coefficient of the lateral component. Therefore, the forces point toward the opposite side of segmental velocities and are nearly perpendicular to the longitudinal axes of the segments. This results in a similar pattern of body forces in the granular medium when compared to movement in fluid or on the surface of the ground [9], [41]. For example, larger thrust forces are generated from the segments with larger angles relative to the forward direction. The oscillations in the three degrees of body motion and the patterns for the amplitude of lateral displacement are all similar to those from a computational study of swimming in a fluid [42]. Similar to swimming in low Re fluids, inertial forces are negligible for swimming in the granular medium, and a steady swimming state is reached within a fraction of a cycle.

The different force relations do, however, lead to differences in the swimming mechanics. Due to the nearly speed-independent resistive forces, neither undulation frequency nor undulation amplitude greatly influences the magnitude of force on the body of a swimmer in the granular medium. In contrast, in true fluids, forces depend on speed. Because of the speed independence in granular media, power consumption increases linearly with frequency, and cost of transport is independent of frequency. This implies there is no optimal frequency for swimming in a granular medium in terms of mechanical energy cost, assuming energy cost associated with accelerating the body/segments is negligible. In true fluids, power typically scales with speed with a power greater than unity and mechanical cost of transport depends on frequency. Unlike true fluids, granular media in the frictional fluid regime exhibit a hysteresis effect because thermal fluctuations do not destroy initial states or disturbed states. In high Re fluids, forces on a swimmer can also depend on the history of the swimmer's motion, but these are typically due to the relatively slow decay of the fluid flow in time [11]. To utilize unsteady hydrodynamic forces, swimmers in high Re fluids often propel themselves at preferred frequencies [9], [43]. Previous studies and theory for swimming in high Re fluids (e.g [9], [43]) suggest that better momentum transfer (greater thrust from inertia) can be achieved by using posteriorly increasing amplitudes of undulation. We hypothesize that the main reason for the insignificant increase of amplitude along the body of the sandfish is because inertial forces are negligible during sand-swimming.

In conclusion, using models developed in previous studies to predict average swimming speed and optimal kinematics for swimming speed, here we have analyzed for the first time the detailed mechanics of undulatory swimming in a friction dominated granular fluid. Our study reveals features of sand-swimming that are particular to granular media, such as speed insensitive resistive forces and therefore speed insensitive mechanical CoT. The simulation demonstrates that head drag is important in determining the motion of the sand-swimmer and consumes significant energy. The simulation allowed us to examine the domain of validity for the empirical force relations used in the RFT model of sand-swimming: The force distribution on the swimmer was approximated well by the empirical force relations, although the transient effect during the starting and reversing of motion in granular drag must be considered to create more accurate force relations. Our model also generates testable predictions for biological sand-swimming. For example, we predict that sand-swimming is energetically demanding and swimming speed may be constrained by muscle physiology, which could limit the use of sustained sand-swimming for long distance travel. The simulation also allows comparison of undulatory locomotion in a granular medium to swimming in true fluids. For example, the similar “w”-shaped pattern of the lateral motion of segments and bell-shaped power and torque distributions compared to previous studies in fluids suggest these patterns are general for undulatory locomotion.
